# Small Intestinal Neuroendocrine Tumor Presenting as Mechanical Bowel Obstruction in an Elderly Patient: A Case Report

**DOI:** 10.7759/cureus.91540

**Published:** 2025-09-03

**Authors:** Mark Salib, John Salib, George Francis, Tamir B Mohamed, Frederick Tiesenga

**Affiliations:** 1 School of Medicine, St. George's University School of Medicine, St. George's, GRD; 2 Surgery, University of Illinois Chicago, Chicago, USA; 3 General Surgery, West Suburban Medical Center, Chicago, USA

**Keywords:** abnormal findings, case report, general surgery, mesenteric tumor, neuroendocrine cell tumor

## Abstract

Gastrointestinal neuroendocrine tumors (NETs) are an uncommon malignancy that often goes undiagnosed until they cause significant symptoms. They are characterized by numerous NET markers, which are helpful for both diagnosing and monitoring treatment in these patients. In this case report, we present an 81-year-old woman with a three-week history of intermittent diffuse abdominal pain. An abdominal CT scan demonstrated abnormal abdominal wall thickening. Surgical resection confirmed a well-differentiated NET. This case report underscores the significance of clinical suspicion, diagnostic accuracy, and the vital role of surgical resection in preventing disease progression and severe complications.

## Introduction

Neuroendocrine tumors (NETs) are slow-growing heterogeneous masses. They can arise from different organ systems in the body, the most common being the gastrointestinal tract, lungs, pancreas, and thyroid gland [1]. These tumors are primarily composed of neuroendocrine cells, which contain both neural and endocrine components [2]. They can receive neural stimulation from the nervous system and convert it into endocrine signals, such as the production of peptides, amines, and other hormones [1,2]. Patients tend to be primarily asymptomatic but may develop symptoms as the tumor enlarges, including diffuse abdominal pain, weight loss, abdominal masses, bowel obstruction, or perforation. The treatment and prognosis depend broadly on tumor grade, location, and stage at diagnosis [3,4].

We present a case of an 81-year-old woman who came to the emergency department presenting with nonspecific abdominal pain and a small bowel obstruction. The diagnosis was established through imaging and confirmed surgically and histologically. This case illustrates the diagnostic challenges of NETs and highlights the role of surgery in managing localized disease.

## Case presentation

An 81-year-old woman presented to the emergency room with a three-week history of intermittent, diffuse abdominal pain that had progressively worsened in frequency and intensity. She described the pain as crampy, non-radiating, and exacerbated after meals, with partial but temporary relief after episodes of emesis. Her appetite had significantly diminished, and she reported early satiety. Over the same period, she noted decreased frequency of bowel movements and reduced passage of flatus, along with several episodes of non-bloody, non-bilious vomiting. Her review of systems was negative for shortness of breath, cough, chest pain, palpitations, dysuria, hematuria, flank pain, or recent travel. She denied recent sick contacts, new medications, or recent hospitalizations. Past medical history was significant only for psoriasis. She denied prior abdominal surgeries, inflammatory bowel disease, or malignancy. She reported no known drug allergies. She lived independently, was a lifelong non-smoker, consumed alcohol rarely, and denied illicit drug use.

On examination, the patient appeared well-kept but fatigued and in mild distress due to abdominal discomfort. She was alert, oriented to person, place, and time, and conversant. Vital signs were within normal limits. Abdominal examination revealed a soft, nondistended abdomen with tenderness to both superficial and deep palpation in the periumbilical region and left lower quadrant. No rebound tenderness, rigidity, or guarding was appreciated. There were no palpable masses or organomegaly. Bowel sounds were present but hypoactive. There was no costovertebral angle tenderness. Both McBurney’s and Murphy’s signs were negative.

On admission, the complete blood count revealed a mild normocytic anemia, with hemoglobin of 11.9 g/dL and hematocrit of 35.6%, marginally below the lower reference range. This finding may be attributable to chronic disease, nutritional deficiency, or anemia of inflammation. The total white blood cell count was within normal limits; however, a relative neutrophilia (73.7%) and mild lymphopenia (16.7%) were observed, findings that may reflect a physiologic stress response or evolving inflammatory process. Platelet count was normal. The comprehensive metabolic panel demonstrated normal electrolyte balance, renal function, and glucose levels. Liver function tests were all within reference ranges. A complete summary of laboratory values is provided in Table 1.

**Table 1:** Laboratory results on admission. Results demonstrate mild anemia, normal leukocyte count with relative neutrophilia and lymphopenia, and preserved renal, hepatic, and metabolic function. Overall, findings indicate intact end-organ function with only mild hematologic abnormalities.

Category	Test	Result	Reference Range
Comprehensive Metabolic Panel (CMP)	Sodium (Na)	134 mmol/L	135 – 145 mmol/L
	Potassium (K)	4.0 mmol/L	3.5 – 5.0 mmol/L
	Chloride (Cl)	102 mmol/L	98 – 107 mmol/L
	Carbon Dioxide (CO₂)	26 mmol/L	22 – 29 mmol/L
	Blood Urea Nitrogen (BUN)	3 mg/dL	7 – 20 mg/dL
	Creatinine (Cr)	0.65 mg/dL	0.6 – 1.3 mg/dL
	BUN/Creatinine Ratio	6	10 – 20
	Glucose	83 mg/dL	70 – 110 mg/dL
	Calcium (Ca)	8.2 mg/dL	8.5 – 10.5 mg/dL
	Total Protein	5.3 g/dL	6.0 – 8.3 g/dL
	Albumin (Alb)	2.8 g/dL	3.5 – 5.0 g/dL
Liver Function Test (LFT)	Aspartate Aminotransferase (AST)	17 U/L	10 – 40 U/L
	Alanine Aminotransferase (ALT)	17 U/L	7 – 56 U/L
	Alkaline Phosphatase (ALP)	92 U/L	44 – 147 U/L
	Total Bilirubin (Tbili)	0.3 mg/dL	0.1 – 1.2 mg/dL
Complete Blood Count (CBC)	White Blood Cell Count (WBC)	6.6 ×10³/µL	4.0 – 10.5 ×10³/µL
	Red Blood Cell Count (RBC)	3.40 ×10⁶/µL	4.2 – 5.4 ×10⁶/µL
	Hemoglobin (Hb)	11.9 g/dL	12.0 – 16.0 g/dL
	Hematocrit (Hct)	35.6 %	36 – 46 %
	Mean Corpuscular Volume (MCV)	83.8 fL	80 – 100 fL
	Mean Corpuscular Hemoglobin (MCH)	28.4 pg	27 – 33 pg
	Mean Corpuscular Hemoglobin Concentration (MCHC)	33.9 g/dL	32 – 36 g/dL
	Red Cell Distribution Width (RDW)	15.2 %	11.5 – 14.5 %
	Platelets (Plt)	426 ×10³/µL	150 – 450 ×10³/µL
	Mean Platelet Volume (MPV)	7.5 fL	7.5 – 11.5 fL
Complete Blood Count (CBC) with Differential	Neutrophils %	73.7 %	40 – 70 %
	Lymphocytes %	16.7 %	20 – 40 %
	Monocytes %	5.1 %	2 – 8 %
	Eosinophils %	3.9 %	1 – 4 %
	Basophils %	0.6 %	0 – 1 %
	Neutrophils Absolute	4.9 ×10³/µL	1.8 – 7.5 ×10³/µL
	Lymphocytes Absolute	1.1 ×10³/µL	1.0 – 4.0 ×10³/µL
	Monocytes Absolute	0.3 ×10³/µL	0.2 – 0.8 ×10³/µL
	Eosinophils Absolute	0.3 ×10³/µL	0.0 – 0.5 ×10³/µL
	Basophils Absolute	0.0 ×10³/µL	0.0 – 0.1 ×10³/µL

**Figure 1:**
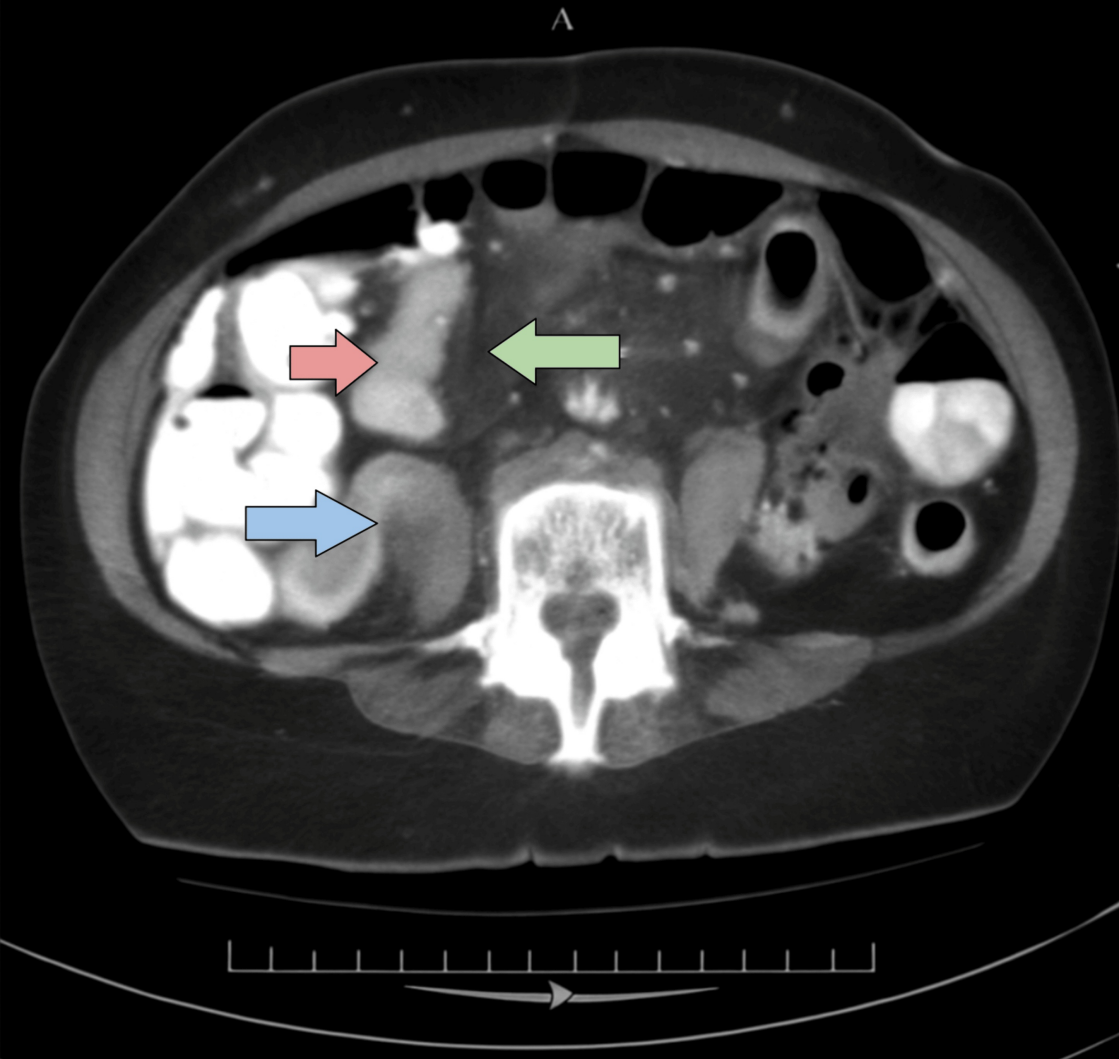
Axial contrast-enhanced CT scan of the abdomen. Axial contrast-enhanced computed tomography of the abdomen demonstrating mechanical small bowel obstruction. The red arrow indicates air-fluid levels within a proximal small bowel loop. The blue arrow highlights a dilated small bowel segment, while the green arrow marks the transition point between the dilated proximal and collapsed distal bowel in the mid-abdomen. No radiologic signs of ischemia or perforation are evident.

A plain abdominal radiograph demonstrated multiple air-fluid levels consistent with small bowel obstruction. Contrast-enhanced CT of the abdomen revealed focal small bowel wall thickening accompanied by prominent mesenteric lymphadenopathy (Figure 1, arrows highlight the affected bowel segment and lymph nodes).

**Figure 2:**
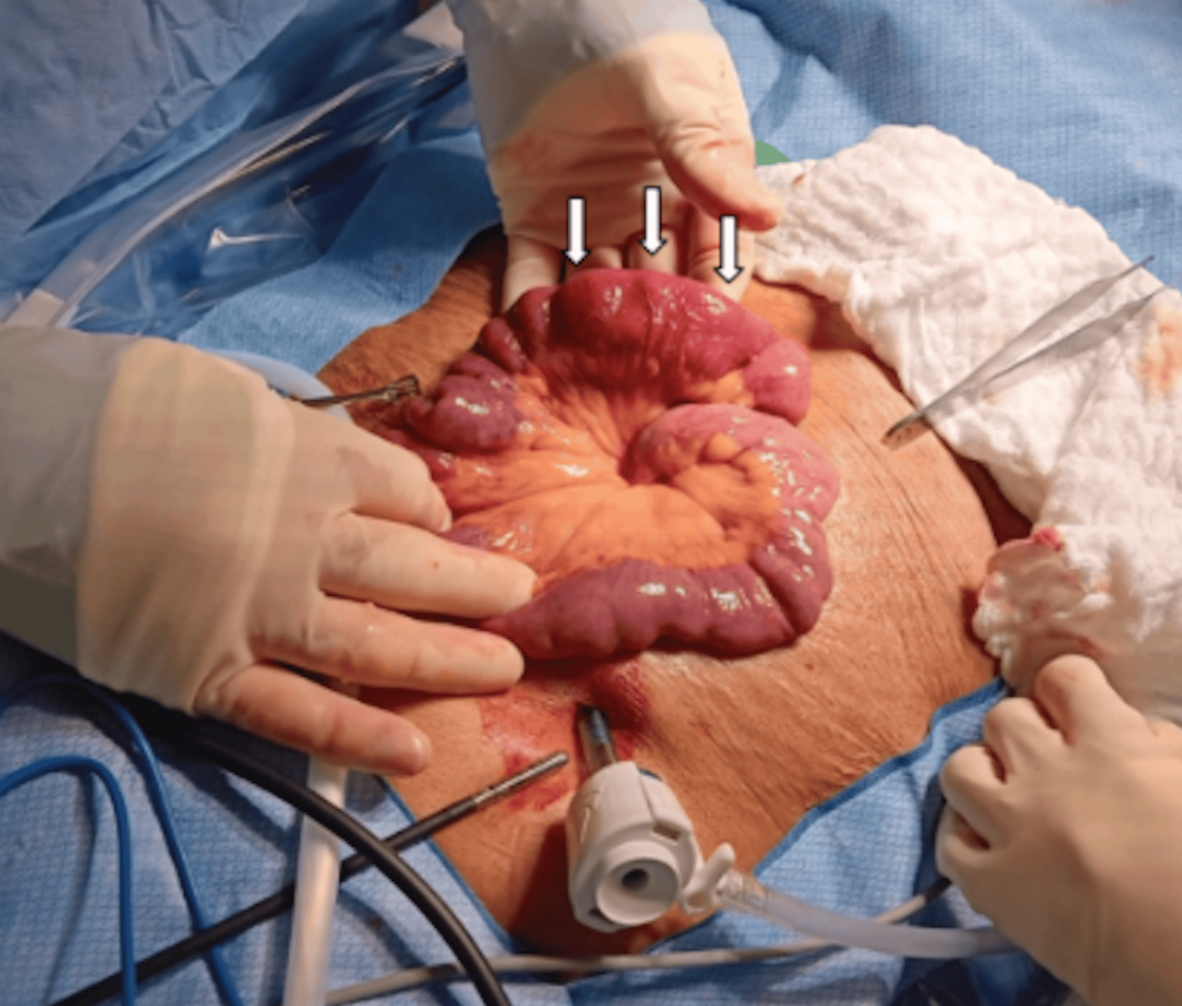
Intraoperative identification of a small bowel transition zone in suspected bowel obstruction. Intraoperative image obtained during diagnostic laparotomy demonstrating a segment of small intestine. The arrows identify the transition zone, where dilated proximal bowel loops abruptly taper into collapsed distal segments, consistent with a mechanical small bowel obstruction. An irregular, thickened, and dusky segment of the small intestine is also evident, corresponding to the area of concern. Intraoperatively, a firm mass was palpated at this abnormal segment.

The patient was initially managed conservatively with bowel rest, intravenous fluids, and broad-spectrum antibiotics for four days. Despite this, she continued to experience abdominal pain, nausea, vomiting, and failure to tolerate oral intake, consistent with persistent obstructive symptoms. Given the lack of improvement, surgical intervention was pursued. A diagnostic laparoscopy revealed ischemic segments of the small bowel and extensive mesenteric lymphadenopathy (Figure 2). A small bowel resection was performed, and continuity was restored using a gastrointestinal anastomosis (GIA) stapler. The common channel was closed with a GIA stapler, and a reinforcing crotch stitch was placed with silk suture. Meticulous hemostasis was ensured, the mesenteric defect was closed, and the anastomosis was tested under pressure, confirming airtight integrity. The specimen was submitted for pathological examination.

**Figure 3:**
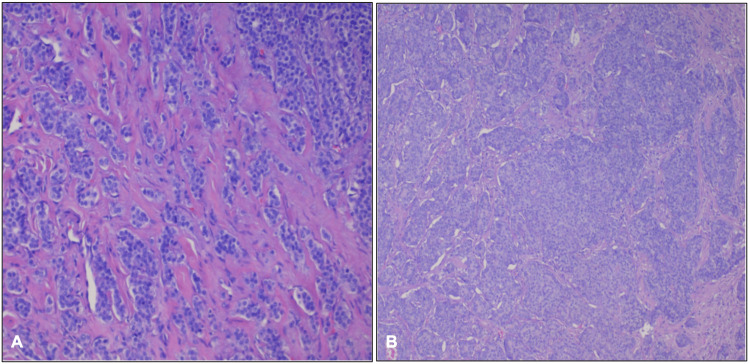
Histopathological examination of resected bowel segment. A: higher magnification (H&E, ×40); B: lower magnification (H&E, ×10) Histopathological examination of the resected bowel segment, stained with hematoxylin and eosin (H&E), demonstrated a well-differentiated neuroendocrine tumor (NET). The lesion was composed of uniform tumor cells arranged in glandular and tubular patterns. The cells exhibited round to oval nuclei with finely dispersed chromatin and scant cytoplasm. No necrosis or appreciable mitotic activity was observed, consistent with a low-grade histological classification.

Tissue samples from the resected bowel were submitted for pathological analysis. Histology demonstrated relatively uniform tumor cells infiltrating the small bowel wall, arranged predominantly in glandular and tubular patterns (Figure 3). Immunohistochemistry showed diffuse positivity for synaptophysin, chromogranin, and pancytokeratin (AE1/AE3), with negative staining for S-100. Ki-67 proliferation index was <1%. These features are consistent with a well-differentiated, grade 1 neuroendocrine tumour.

## Discussion

Intestinal NETs are rare, indolent malignancies derived from hormone-producing cells of the gastrointestinal tract. While most cases are sporadic, a minority are associated with hereditary syndromes such as multiple endocrine neoplasia type 1 (MEN1) [5-7]. Clinical manifestations vary depending on the tumor location and hormonal activity. Functional NETs may produce carcinoid syndrome, characterized by diarrhea, flushing, wheezing, and valvular heart disease, whereas nonfunctional tumors typically remain asymptomatic until they cause local complications or metastasis [8,9].

The presentation of small bowel obstruction secondary to an intestinal NET is uncommon and may mimic several other gastrointestinal pathologies. As depicted in Table 2, adhesions, hernias, inflammatory bowel disease, or adenocarcinoma of the small intestine are more frequently associated with mechanical obstruction. Clinical features such as abdominal pain, distension, nausea, and vomiting are nonspecific, making it challenging to distinguish NETs without advanced imaging or histopathology. In particular, adenocarcinomas may present with localized thickening and obstruction, while Crohn’s disease can produce segmental bowel wall thickening and strictures. This overlap highlights the importance of considering NETs in the differential diagnosis of small bowel obstruction, especially when routine etiologies are not apparent.

**Table 2:** Differential diagnoses of small bowel obstruction with overlapping features. This table compares common causes of small bowel obstruction that may mimic the presentation of an intestinal neuroendocrine tumor (NET). While conditions such as adhesions, hernias, adenocarcinoma, and Crohn’s disease share overlapping symptoms and imaging findings, NETs can be distinguished by their neuroendocrine origin and positive immunohistochemical markers (chromogranin A, synaptophysin, pancytokeratin).

Condition	Typical Features	Similarity to NET Presentation	Distinguishing Features
Adhesions	History of prior surgery; acute obstruction	Abdominal pain, distension	No primary mass on imaging
Hernia	Palpable mass, localized tenderness	Mechanical obstruction	External or internal defect visible
Small bowel adenocarcinoma	Localized wall thickening, obstruction	Mass effect, obstruction	More aggressive, earlier systemic symptoms
Crohn’s disease	Chronic diarrhea, weight loss, strictures	Segmental thickening, obstruction	Inflammatory markers, mucosal ulceration
NET (nonfunctional)	Often asymptomatic until obstruction	Local thickening, transition point	Positive neuroendocrine markers (Chromogranin A, Synaptophysin)

Accurate diagnosis of intestinal NETs requires integration of imaging with histopathology. Contrast-enhanced CT remains the preferred modality for initial detection and staging, but histological confirmation is essential. Immunohistochemical (IHC) staining provides diagnostic specificity, with chromogranin A, synaptophysin, and pancytokeratin serving as hallmark markers [10,11]. In our case, diffuse positivity for chromogranin A, synaptophysin, and pancytokeratin (AE1/AE3) established the neuroendocrine origin of the lesion, effectively ruling in NET as the underlying cause of obstruction. The absence of necrosis and mitotic activity further supported a low-grade tumor classification, aligning with the indolent behavior often observed in these neoplasms. Serum chromogranin A, while commonly used as a biomarker for diagnosis and monitoring, was not contributory in this acute presentation, highlighting the indispensable role of tissue confirmation.

Management of intestinal NETs is complex and requires a multidisciplinary approach involving surgical oncology, medical oncology, radiology, and gastroenterology. For localized disease, surgical resection remains the cornerstone of treatment and the only potentially curative modality [11]. Resection typically involves the removal of the primary tumor with adequate margins and regional lymphadenectomy, as nodal involvement is common even in early disease. In our patient, resection was performed urgently due to bowel obstruction, underscoring that acute complications often dictate the timing and extent of surgery.

For patients with metastatic disease, management strategies may extend beyond surgery. Cytoreductive procedures, somatostatin analogs (e.g., octreotide, lanreotide), targeted therapies (e.g., everolimus, sunitinib), and peptide receptor radionuclide therapy (PRRT) are established options that improve symptom control and progression-free survival [9,11]. The choice of therapy is influenced by tumor burden, functional status, and patient comorbidities. Importantly, even in the presence of metastatic disease, resection of the primary tumor is often considered, as it reduces the risk of future obstruction, ischemia, and bleeding.

This case illustrates the importance of early recognition and surgical intervention in patients with intestinal NETs. Although our patient lacked the hormonal features typical of functional tumors, timely operative management prevented further complications and facilitated a definitive diagnosis. Clinicians should therefore consider NETs in the differential diagnosis of unexplained small bowel obstruction, particularly when imaging reveals localized thickening without a clear alternative etiology.

## Conclusions

This case highlights an uncommon presentation of an intestinal NET manifesting as small bowel obstruction in the absence of hormonal symptoms. NETs are often indolent and clinically silent until advanced, making early recognition challenging. As demonstrated here, their presentation can mimic more common causes of obstruction, such as adhesions, hernias, adenocarcinoma, or Crohn’s disease. A definitive diagnosis requires histopathologic confirmation with characteristic immunohistochemical staining, as seen in this patient, who exhibits diffuse positivity for chromogranin A, synaptophysin, and pancytokeratin. Management remains multidisciplinary, with surgical resection as the cornerstone of therapy for localized disease. In this case, timely operative intervention not only relieved the obstruction but also enabled definitive diagnosis and staging. Clinicians should therefore maintain a high index of suspicion for NETs in patients with unexplained small bowel obstruction, as early identification and prompt surgical management are critical for optimizing outcomes.
